# The role and application of internal and external load indicators in monitoring fatigue in rugby players—a narrative review

**DOI:** 10.3389/fspor.2025.1654577

**Published:** 2025-09-24

**Authors:** Xuehaiyue Lv, Jingyuan Yang, Lintao Suo, Wei Han

**Affiliations:** ^1^ Graduate School of Shandong Sport University, Shandong Sport University, Jinan, China; ^2^ College of Competitive Sports, Shandong Sport University, Jinan, China

**Keywords:** load management, overtraining, recovery, performance, team sport

## Abstract

Although the scientific understanding of training load and fatigue monitoring in rugby has advanced considerably, critical challenges remain in the systematic implementation and integrative interpretation of internal and external load metrics. Through a comprehensive narrative review of PubMed, Web of Science, and CNKI databases encompassing literature through December 2024, we critically examined the application and limitations of multiple monitoring approaches including GPS and inertial microsensor technologies, time-motion analysis, neuromuscular function assessments, subjective rating scales, cardiac autonomic markers, and biochemical profiling. Our synthesis reveals substantial methodological heterogeneity and lack of consensus regarding optimal implementation strategies for these metrics in rugby-specific contexts. Moving forward, research efforts should prioritize three key areas: (1) developing sport-specific algorithms for multi-modal data integration that account for rugby's unique physical demands; (2) establishing individualized monitoring protocols that consider positional requirements and athlete characteristics; and (3) validating predictive models that enhance the precision of load quantification while supporting evidence-based training prescription and injury risk mitigation in competitive rugby environments. These advancements would address current limitations in monitoring practice and provide practitioners with more reliable tools for optimizing athlete preparation and performance outcomes.

## 1 Introduction

Rugby is a high-intensity, full-contact team sport that demands players execute diverse athletic movements-including change-of-direction sprints, tackles, scrums, and ball handling—interspersed with brief recovery periods (1–3). To meet these physical challenges, players must develop comprehensive physical capacities encompassing maximal strength, power, speed, agility, and both aerobic and anaerobic fitness (4). Training induces both fitness gains and fatigue accumulation, with net performance determined by their balance, making the monitoring of these adaptations particularly valuable in rugby—a sport requiring repeated high-intensity efforts where performance is influenced by both physiological capacity and external match factors (5).

The extended duration and increased fixture density of modern rugby seasons (rugby league, rugby union, and rugby sevens) (5, 6). Intensive training and matches can lead to cumulative neuromuscular fatigue, metabolic disruption, and immune system suppression in athletes, thereby significantly impairing performance and increasing the risk of acute injuries by 1.6–2.3 times (7). Research has confirmed that successful training programs must incorporate overload work but must also avoid the combination of excessive overload and insufficient recovery (8, 9). Therefore, timely and systematic fatigue monitoring is essential for rugby players.

Currently, research on fatigue monitoring following training sessions and matches primarily relies on the measurement of various indicators, such as neuromuscular function, physiological and biochemical parameters, heart rate, perceptual responses, GPS data, and information from 3D sensors. Most studies report on multiple load monitoring metrics (8–10). However, a consensus has yet to be reached regarding the temporal patterns of recovery following training and matches, as well as the optimal application of these measurement indicators. Additionally, given that diverse training programs, distinct training models, and varied recovery protocols can lead to disparate research outcomes, the extent and duration of recovery may also differ, which could in turn impact the practical applicability of the data.

The purpose of this review is to provide a comprehensive synthesis of the existing literature to (a) quantify the magnitude and temporal dynamics of responses following intensity-based stimuli; (b) elucidate the roles and application values of load monitoring indicators before and after training and competition, and analyze the limitations of different monitoring indicators in this context; (c) optimize load-recovery balance strategies, provide theoretical support, and thereby effectively mitigate the risk of overtraining, maintain and enhance athletic performance, and reduce the incidence of sports injuries.

## 2 Methods

A comprehensive literature search was conducted through PubMed, Web of Science, and China National Knowledge Infrastructure (CNKI) up to December 2024. Search terms included the following keywords: “rugby”, “rugby union”, “rugby league”, “rugby sevens”, “load monitoring”, “external load”, “mechanical load”, “physical demands”, “internal load”, “physiological stress”, “metabolic load”, “autonomic response”, “neuromuscular fatigue”, “fatigue”, “overtraining”, “exercise-induced fatigue”, “recovery”, “post-exercise recovery” which were combined with Boolean operators (“AND” and “OR”). A supplementary search was conducted in Google Scholar to identify additional studies not captured by the primary database searches. Literature lists of articles and books were also downloaded.

This narrative review approach was selected to provide a comprehensive synthesis of current knowledge while allowing flexible examination of emerging trends and literature gaps (20). The study selection process prioritized articles relevant to rugby-specific load monitoring, incorporating both English and Chinese literature to ensure broad representation of regional perspectives and technological developments in the field.

Initial database searches were supplemented by manual screening of reference lists from key articles, with all identified studies systematically organized using EndNote 20. The inclusion criteria required studies to: involve rugby players at any competitive level; specifically examine internal or external load monitoring methods; be available as full-text publications in English or Chinese; and appear in peer-reviewed journals or credible conference proceedings. Studies were excluded if they lacked quantitative or qualitative load monitoring data, focused on non-contact sports, or represented duplicate publications.

## 3 Load monitoring based on automated technology

In the field of sports training, external load monitoring quantifies athletes' training workloads to assess training stress, and its correlation with athletic performance has been well established (1, 9–13). Traditional approaches to training load monitoring have primarily relied on static physiological indicators obtained prior to competition and retrospective analyses (12). However, the advent of modern monitoring technologies has revolutionized this field by enabling the dynamic integration of training load indicators, thereby significantly enhancing the accuracy and timeliness of load monitoring.

In the context of rugby, the integration of Global Positioning Systems (GPS) with various sensors—such as accelerometers, gyroscopes, and magnetometers—permits the real-time acquisition of data pertaining to athletes' positions, speeds, accelerations, and changes in direction. This wealth of data provides robust support for the analysis of movement patterns (13). Despite its efficacy in quantifying movement patterns and external load in rugby match play, GPS technology is limited by sampling frequency (e.g., 10 Hz), signal interference, and its inability to directly capture biomechanical details (such as changes of direction and contact actions) (9, 11). Therefore, integrating multimodal data is essential to enhance the reliability of analysis (9, 11) (Table 1).

**Table 1:** Summary of key findings on training load in rugby players using automated technologies.

Authors	Year	Study objectives	Main results	Reference
Billy et al.	2016	Collision frequency during rugby league matches is associated with team success, fatigue, and injury risk. This study investigates the sensitivity and specificity of wearable microtechnology to identify collision events during professional rugby league matches.	Microtechnology can identify 97.6% of collision events during rugby league match-play.The typical error associated with measuring contact events can be reduced to 7.8%, with accuracy(92.7%) and specificity (91.7%) improving, when low-intensity (<1 PlayerLoad AU) and short duration(<1 s) collision reports are excluded.	(14)
Shane et al.	2019	This study reviews the movement demands of rugby sevens using GPS technology. Key findings include male players covering greater relative distance and having higher maximum velocities compared to females.	Relative distance in the first half of matches is higher than that in the second half of matches for all sexes and levels of competition. GPS also tracks high-intensity running, accelerations, decelerations, and impacts.The study highlights the need for sex-specific training and fatigue management strategies.	(15)
Dalton-Barron et al.	2021	This study analyzes match-running variability in elite rugby league using microtechnology data from 322 games.	Key findings show large within-and between-player variability in total distance and HSR, while average speed and acceleration remain stable, The transition phase exhibits highest variability.Results suggest players self-regulate speed consistently despite phase-specific demands, with absolute displacement metrics being more variable.	(18)
Naughton et al.	2021	To evaluate how post-match recovery has been quantified in rugby codes (rugby league, rugby union, and rugby sevens). explore the time-course of commonly used fatigue measures post-match. Investigate the relationships between game-related collisions and fatigue metrics.	Regarding collision intensity, microtechnology devices report substantial differences in the frequency of mild, moderate, and heavy collisions, with a notable lack of consistency across studies. Microtechnology devices have not been adequately validated in quantifying collision frequency and intensity, and thus have certain limitations.	(21)
Collins et al.	2023	Significant differences in movement patterns were observed between international and domestic competitions, with international matches featuring higher occurrences of high-velocity movement units.	This indicates that international rugby league players may experience greater high-velocity demands, highlighting the need for tailored training programs. The study demonstrates that the SMP framework can effectively differentiate between competition levels and offers a detailed understanding of movement demands in rugby league, enhancing training specificity and athlete preparation.	(11)

GPS, global positioning systems; SMP, sequential movement pattern; HSR, high-speed running.

### 3.1 Load monitoring based on GPS and microsensor technology

Research has demonstrated that GPS technology can effectively monitor training load through the acute: chronic workload ratio (ACWR) (14). In rugby, this technology reveals distinct load characteristics among players in different positions. For instance, forwards, who undertake more defensive tasks, experience a collision frequency of 18.3–44.0 per match, significantly higher than the 26.0 collisions per match recorded by backs (13, 14). During rugby 7's matches, relative distance in the first half of matches is higher than that in the second half of matches for all sexes and levels of competition (15). Additionally, by integrating a triaxial accelerometer with GPS devices (16), the system can calculate a body load index (Body Load, AU) based on changes in axial acceleration (x/y/z) and body weight parameters (14, 17, 18).

The ACWR, calculated via methods such as coupled/uncoupled rolling averages (RA) and exponentially weighted moving averages (EWMA), provides valuable insights into workload management, with minimal practical differences observed between these methods despite their theoretical distinctions (9). Notably, workload monitoring must also account for high-intensity collision demands—a unique feature of rugby. Research indicates that a single high-intensity collision can induce acute physiological stress, particularly during sudden stops, resulting in a 28% increase in peak blood lactate and a 45% rise in creatine kinase levels (19). While video analysis was once the primary monitoring method, current microsensor systems using accelerometer-gyroscope combinations have achieved precise quantification of collision load parameters, with results highly consistent with traditional methods (r = 0.96) (14, 20).

However, key technical standards such as sensor placement and impact force threshold determination remain contentious. In training practice, only a minority of coaching staff have integrated these metrics into their decision-making processes, with subjective assessments still dominating. This is primarily due to the fact that traditional monitoring technologies struggle to quantify actions such as isometric efforts (e.g., tackling and grappling), which, despite generating significant metabolic demands, are not directly reflected in kinematic parameters (17). Therefore, future research needs to further optimize microsensor technology to more comprehensively assess the workload of rugby players and provide a scientific basis for training decisions.

### 3.2 Load monitoring based on time-motion analysis

Time-Motion Analysis (TMA) utilizes the Sequential Movement Pattern (SMP) algorithm to dissect rugby movements into seven fundamental states: standing, walking, jogging, running, sprinting, backpedaling, and lateral movement (11, 16). This algorithm further categorizes these movements into 121 composite movement classifications. In practice, TMA can estimate velocity and distance by recording the duration of each movement and applying predefined values associated with each movement category (11). For instance, if a player spends 30 s jogging, the algorithm can estimate the distance covered based on the predefined average jogging speed for that player or team. Alternatively, distance can be estimated by timing an athlete's movement between predetermined visual cues on the field using a stopwatch (velocity = distance/time) (17).

This method can precisely identify movement units, such as sudden stops with changes in direction exceeding 45°, as well as their sequential combinations (20). For example, a sequence might include a player jogging for 10 s, then making a sudden stop and changing direction by 60°, followed by a sprint for 5 s. Studies have shown that in international competitions, athletes exhibit a significantly higher proportion of lateral movements and multidirectional changes compared to national-level matches (13). This difference is primarily attributed to increased tactical complexity and the rate of transitions between attack and defense (11, 17). For example, in international matches, players may need to switch from offense to defense more rapidly, requiring more frequent and sharper directional changes.

### 3.3 Load monitoring based on neuromuscular function

Neuromuscular fatigue represents a fundamental load response in rugby, characterized by exercise-induced reductions in maximal voluntary contraction force (10, 22). Recent investigations have underscored that movement patterns incorporating the stretch-shortening cycle (SSC) can more accurately reflect exercise-specific fatigue (23). Rugby's intermittent nature necessitates repeated acceleration, deceleration, and directional changes—actions demanding rapid eccentric-concentric transitions. Prolonged exposure to these movements impairs SSC efficiency, precipitating neuromuscular fatigue and movement economy deterioration (12) (Table 2).

**Table 2:** Summary of key findings on training load in rugby players using neuromuscular function monitoring technologies.

Authors	Year	Study objectives	Main results	Reference
McLellan et al.	2011	This study investigates the markers of postmatch fatigue in professional rugby league players using automated technology.	Neuromuscular performance, measured via CMJ, showed significant decrements in RFD and peak power up to 48 h postmatch. The findings highlight the utility of automated monitoring tools in assessing player fatigue and guiding recovery strategies.	(23)
Johnston et al.	2015	The study examined the impact of physical qualities on post-match fatigue in rugby league players.	It found that players with better high-intensity running ability and lower-body strength experienced less neuromuscular fatigue, as measured by CMJ and plyometric push-up power.	(29)
Oxendale et al.	2016	This study investigates the relationship between match-play characteristics in elite rugby league and subsequent EIMD using GPS data and neuromuscular function markers.	The study found that match duration, high-intensity running distance, total collisions, and repeated high-intensity efforts were significantly associated with increased CK concentration and decrements in upper-body neuromuscular performance. These findings suggest that recovery strategies should be tailored to individual match demands, as players experiencing higher volumes of these activities exhibit greater muscle damage.	(25)
Dubois et al.	2020	This study examines the impact of weekly workload on neuromuscular function in professional rugby players over a competitive season.	Key metrics include changes in strength, power, and CMJ performance. Results show that chronic workload negatively affects strength and power, with significant correlations between heavy impacts and decreased performance. These findings highlight the importance of managing workload to maintain neuromuscular function and prevent fatigue.	(24)
Naughton et al.	2021	This review examines fatigue in rugby codes, highlighting the crucial role of neuromuscular performance metrics (e.g., countermovement jumpsand plyometric push-ups) in assessing post-match fatigue.	Studies demonstrate significant decrements in peak power, peak force, and rate of force development, these performance declines show strong correlations with collision frequency and intensity, confirming collisions as primary drivers of neuromuscular fatigue.	(21)
McMahon et al.	2021	This study investigates the relationship between RSI variants in rugby league players.	Results show that RSI derived from DJ yields significantly higher values than RSImod from CMJs, with only 22% shared variance. This indicates that the two RSI variants assess different reactive strength qualities and should not be used interchangeably. The findings suggest that DJ-derived RSI is more suitable for monitoring reactive strength due to its higher values and stronger association with neuromuscular function.	(26)

CMJ, countermovement jumps; RFD, rate of force development; EIMD, exercise-induced muscle damage; GPS, global positioning systems; CK, creatine kinase; RSI, reactive strength index; DJ, drop jumps; RSImod, reactive strength index modified.

To monitor neuromuscular function and SSC capability, researchers have employed vertical jump testing protocols, including countermovement jumps (CMJ) and drop jumps (DJ), for quantitative assessment (24). Specifically, the Reactive Strength Index (RSI) and its modified variant (RSImod) serve as key metrics to evaluate SSC efficiency under fatigue conditions. RSI [calculated as jump height divided by ground contact time (GCT) during DJ] reflects fast SSC performance, while RSImod (jump height divided by time to take-off [TTT] in CMJ assesses slow SSC capacity (25–27).

The CMJ test can effectively monitor exercise-induced muscle fatigue in rugby players following matches and training, serving as an indirect indicator of the rate of force development (force development, RFD) (6, 16, 21). By systematically monitoring biomechanical parameters such as peak force (PF) and instantaneous power output (Power Profile, PP) in CMJ tests, combined with changes in RFD, it is possible to effectively track post-exercise fatigue and assess recovery time (10). Studies demonstrate that multiple jumps (i.e., five repeated CMJs) may provide more accurate fatigue monitoring than single jumps, as they can reflect changes in muscle stiffness through posture control and landing impact forces (25, 28).

Research in rugby league players shows RSI and RSImod exhibit only 22% shared variance, indicating they capture distinct neuromuscular qualities and should not be used interchangeably (25). Notably, RSImod is more sensitive to changes in propulsion phase kinetics (e.g., peak force and power) and better discriminates between competitive levels, as evidenced by Super League players achieving higher RSImod through shorter TTT (26, 27). Conversely, DJ-derived RSI depends on rapid force production during brief GCTs (<250 ms), making it valuable for monitoring fast SSC fatigue in high-intensity sports (25, 26). The choice between variants should consider training status: RSImod suits beginners or weaker athletes due to lower mechanical demands, while RSI is optimal for monitoring elite athletes during speed-strength phases (26, 27).

In addition to lower-limb jump tests, upper-limb strength and power are equally critical for rugby players' performance. Research indicates that plyometric push-ups, as a dynamic assessment tool, can precisely evaluate the power output of upper-limb muscle groups by measuring the vertical thrust and movement velocity generated during the exercise (17). Researchers often use portable force platforms to synchronously collect biomechanical parameters such as peak ground reaction force (Fzmax) and rate of RFD, or employ infrared timing systems to measure jump height and contact time, thereby calculating the relative power index (21). Owing to its involvement of the rapid transition mechanism between eccentric and concentric contractions, it has become an effective tool for assessing the recovery of upper-limb neuromuscular function. Johnston (29) found that the power output in plyometric push-ups decreased by 14.6% immediately after a match, by 10.2% compared to baseline after 24 h, and by 4.1% after 48 h. These findings suggest that upper-limb muscle fatigue peaks immediately post-match and recovers more slowly. In contrast, CMJ power can return to baseline levels 24 h after a match, while plyometric push-up power has not fully recovered even after 48 h, further confirming that the recovery process of upper-limb muscles is slower following a match.

Relevant studies have collected data using sensors worn by athletes, categorizing collision impact forces into different intensity ranges. The results showed that moderate-intensity body impacts were significantly negatively correlated with plyometric push-up performance 12 h after the match (30). No significant correlations were found between the frequencies of impacts in other intensity ranges and performance changes (*p* > 0.05). Additionally, in monitoring the movement speed of upper and lower limbs under load (such as squats and bench presses), when the load is below 70% 1RM, the measurement error is small (CV % < 8%), effectively reflecting the post-match fatigue state of athletes (21).

## 4 Internal load monitoring

### 4.1 Training load monitoring based on subjective perceived intensity

The Session-Rating of Perceived Exertion (session-RPE) scale, which was revised by Carl Foster from Borg's RPE scale (0–10), is a convenient and efficient tool for monitoring training load and can accurately measure athletes' subjective feedback on training intensity (31, 32). Its calculation formula [session-RPE (AU) = training duration (min) × RPE score] takes into account not only the external load of training but also athletes' perception of psychological fatigue, and thus has high practical value (32). Data collected through session-RPE can be used to derive key indicators such as weekly training load, training monotony, providing sensitive and reliable assessment of training responses, thereby facilitating performance optimization and overtraining prevention (33, 34), and training intensity, which provide an important basis for scientifically formulating and adjusting training programs (33) (Table 3).

**Table 3:** Summary of key findings on training load in rugby players using subjective perceived intensity monitoring technologies.

Authors	Year	Study objectives	Main results	Reference
Lovell et al.	2013	This study examines the validity of session rating of perceived exertion Session-RPE for monitoring training intensity in rugby league.	It finds significant correlations between Session-RPE and various internal and external measures of training load. A combination of factors, including distance, impacts, and body load, explains a large portion of the variance in sRPE.	(38)
Halson and Shona	2014	Evaluate the effectiveness of subjective perceived intensity monitoring technologies in understanding fatigue and training adaptation in rugby players.	Subjective perceived intensity monitoring technologies, such as session-RPE, are valuable for understanding and managing training load in rugby players. These tools provide practical and effective means to assess internal load and detect fatigue, supporting individualized training adaptations and performance optimization.	(12)
Saw et al.	2016	To systematically review the effectiveness of subjective self-reported measures in monitoring changes in athlete well-being in response to training.	Subjective measures, such as mood states and perceived stress, were found to be more sensitive and consistent in reflecting acute and chronic training loads than objective measures like physiological markers. These findings support the use of subjective self-reported measures as a practical and effective tool for monitoring training load and athlete well-being.	(34)
Blanch and Gabbett	2016	This paper highlights the importance of the acute:chronic workload ratio in assessing injury risk for athletes returning to play.	The study suggests that combining both internal and external load measures provides a more complete picture of an athlete's training load and readiness for competition. The acute:chronic workload ratio should be included in return-to-play decision-making to better manage injury risk.	(39)
Comyns and Hannon	2018	This study investigates how strength and conditioning (S&C) coaches use the session-RPE method to monitor training loads in professional rugby union.	Results show that while most coaches find session-RPE valid and effective, not all adhere to implementation guidelines, potentially limiting data accuracy. Limiting the calculation of cumulative training load, training monotony, and training strain.	(36)
Yu et al.	2021	The session-RPE method offers a simple, non-invasive approach to quantify internal training load, validated across various sports and populations.	The method helps prevent overtraining and injury by tracking ACWR. Despite its reliability, session-RPE has limitations, such as subjectivity and variability in reporting. Combining it with objective measures like heart rate can enhance accuracy.	(32)

Session-RPE, session rating of perceived exertion; ACWR, acute: chronic workload ratios.

Session-RPE are logistically feasible in rugby training or competition settings, as they minimally disrupt training schedules while allowing for adequate athlete recovery and practical implementation (35, 36). The timing of Session-RPE scale assessments can be tailored to the specific demands of the training session and individual athlete characteristics (37).Short-term monitoring is valuable for evaluating acute fatigue, whereas longitudinal tracking better reflects recovery status (36, 37).

Training impulse—a more nuanced understanding of the variability in session-RPE-TL (training load based on session-RPE) can be achieved. In rugby training, different training session intensities (such as resistance training, physical conditioning, and technical training) significantly influence session-RPE scores. Physical conditioning sessions often yield higher session-RPE values, averaging 8.8 ± 1.1. In contrast, external load factors such as acceleration sprints and total running distance are closely related to athletes' subjective perceptions (38), while internal load factors (such as percentage of heart rate and training impulse) have relatively smaller impacts on session-RPE (36).

When employing session-RPE as a monitoring indicator, coaches and researchers should emphasize the systematic collection and analysis of data. For instance, the Acute: Chronic Workload Ratio (ACWR) quantifies injury risk by comparing recent (1-week) and baseline (4-week average) training loads (36, 39). An ACWR of 0.8–1.3 suggests optimal load management with lower injury risk, while values >1.5 indicate heightened injury susceptibility due to rapid load spikes (39). In collision sports, ACWR effectively monitors high-impact exposure (e.g., sudden increases in contact frequency), where ratios exceeding 1.5 signal elevated injury risk from abrupt workload changes (36, 39).

These findings provide a scientific basis for monitoring and adjusting training load, further demonstrating the role of session-RPE in quantifying training load and assessing exercise fatigue. To gain a multidimensional understanding of the impact of training on athletes' physical performance in rugby, some studies have suggested using differential RPE (dRPE), which separates subjective load into separate assessments for the legs, upper limbs, and cardiovascular system. Compared with session-RPE, dRPE appears to offer a more comprehensive and nuanced method of load monitoring, capable of more accurately reflecting athletes' actual training and match loads (10).

In summary, while the session-RPE method is operationally straightforward, its limitations should not be overlooked. Balancing workloads across different training modes requires consideration of the physiological characteristics and individual differences of each mode. Additionally, the current lack of a validated unified indicator to quantify external training load limits comparisons between different training modes and may introduce biases in research outcomes (33, 34). Therefore, future research needs to further explore ways to optimize quantification methods to enhance the accuracy of training load assessment.

### 4.2 Training load monitoring based on heart rate

Heart rate monitoring is a versatile and multifaceted physiological surveillance method and serves as a crucial indicator for tracking training load (40). Given the linear relationship between heart rate and oxygen consumption, heart rate data can effectively reflect exercise intensity (41, 42). These data can promptly identify signs of overtraining, thereby allowing for adjustments to training programs to facilitate recovery24 (40–42). By concurrently monitoring athletes' external loads and internal physiological responses, one can accurately assess the impact of training intensity on physiological adaptation (17, 25) (Table 4).

**Table 4:** Summary of key findings on training load in rugby players using heart rate monitoring technologies.

Authors	Year	Study objectives	Main results	Reference
Weaving et al.	2014	This study investigates the dose–response relationship between training load and aerobic fitness in academy rugby union players, it finds that iTRIMP explains changes in VO_2_max.	These internal training load measures display curvilinear relationships with changes in maximal aerobic fitness, suggesting that excessive training loads may lead to decreased aerobic fitness. The study highlights the importance of using appropriate heart rate–based training load measures to optimize training outcomes.	(53)
Bellenger et al.	2016	This systematic review and meta-analysis examines the effect of training interventions on autonomic heart rate regulation in athletes.	Resting and post-exercise HRV, post-exercise HRR, and heart rate acceleration increase with positive training adaptations. However, these metrics also increase in response to overreaching training, complicating their interpretation. HR acceleration appears to decrease with overreaching, suggesting it may be a useful marker of training-induced fatigue.	(40)
Schneider et al.	2018	The study aims to outline the current limitations and applications of heart rate monitoring in team sports, provide a conceptual framework for contextualizing HR measures to optimize training and recovery.	Heart rate monitoring is a valuable tool for understanding and managing training load in rugby players. By contextualizing HR measures and using multivariate analysis, practitioners can make informed decisions to optimize training and recovery, ultimately enhancing performance and reducing injury risk.	(43)
Scott et al	2018	This study examines the reliability and usefulness of a SSRIndiv for monitoring fitness and fatigue in elite rugby league players.	Results show acceptable reliability for HRex and HRR during the test. The SSRIndiv can detect true and substantial individual changes in HRex and HRR with precision.	(47)
Andrew and Howells	2018	This study evaluates the weekly HRV responses to varying training loads among an Olympic rugby sevens team.	Players with smaller fluctuations in LnRMSSDcv during intensified training showed more favorable changes in running performance, suggesting that HRV can be a useful tool for monitoring training adaptations.	(61)
Flatt et al.	2019	This study evaluated the effects of varying training load on HRV and running performance in an Olympic rugby sevens team.	Results showed that the LnRMSSDcv increased slightly after the first week of intensified training and then decreased moderately in the third week. Players with smaller fluctuations in HRV during intensified training demonstrated more favorable changes in running performance.	(44)
Grainger et al.	2022	This study examines the autonomic nervous system indices of player readiness during elite-level rugby union game-week microcycles.	The study identifies significant reductions in HRV post-match, indicating disturbed autonomic control, but notes that HRV recovers quickly within the weekly microcycle.	(60)

HRV, heart rate variability; HRR, heart rate recovery; HR, heart rate; LnRMSSDcv, coefficient of variation of HRV; HRex, exercise heart rate; SSRIndiv, submaximal shuttle run test; iTRIMP, individualized training impulse; HRV, heart rate variability.

#### 4.2.1 Monitoring of resting heart rate

The physiological regulation of resting heart rate (RHR) involves multiple mechanisms, including myocardial morphology, plasma volume, autonomic nervous activity, age, and body position. Athletes undergoing high-intensity training or a congested competition schedule often exhibit elevated RHR, with an increase in heart rate during sleep also being observed (43). Standardized measurement of RHR typically requires subjects to lie supine for 10 min or to be taken immediately upon awakening in the morning, with nocturnal RHR being regarded as an ideal indicator for assessing training load intensity due to the exclusion of circadian rhythm interference (44).

However, the effectiveness of RHR as a tool for monitoring training load has yet to reach a consensus in existing research. Some studies have indicated that following intense competitions or training, athletes may develop overtraining syndrome (OTS), which is associated with a significant increase in RHR (40). Nevertheless, other literature suggests that there is no statistically significant difference in RHR between normal training and overtraining states (45). This perspective was elucidated in a systematic review, which found that short-term high-intensity interventions (<2 weeks) can cause a moderate increase in RHR (effect size ES = 0.45), whereas long-term interventions (>2 weeks) show no significant change in RHR (ES = 0.12) (45). These findings suggest that the dynamic changes in RHR are more sensitive to short-term fluctuations in training load but have limited indicative value for long-term training adaptation states.

In rugby, RHR monitoring can serve as an auxiliary indicator of acute fatigue accumulation during short-term high-intensity training or in periods of dense competition schedules. For instance, an increase in RHR can be used as a basis for adjusting athletes' load fatigue responses when there is a sudden increase in weekly training load (17). However, when incorporated into a longitudinal monitoring system throughout an entire season or used to monitor non-functional overreaching and OTS, its sensitivity and specificity are significantly limited (44). Therefore, in training, RHR should be used in conjunction with other monitoring indicators (such as HRV, blood markers) to enhance the accuracy of fatigue assessment.

#### 4.2.2 Monitoring of heart rate during exercise

During training, heart rate (HR)—related metrics such as exercise heart rate (HRex), heart rate reserve (HRres), maximum heart rate (HRmax), and percentage of maximum heart rate (%HRpeak) are widely utilized (42, 46). HRex serves as a crucial indicator for assessing training adaptation and fatigue status. Buchheit's research (42) has demonstrated a strong correlation between HRex and the increase in oxygen uptake (VO2), with changes in HRex reflecting athletes' adaptation to training.

To effectively manage training intensity in elite rugby players, heart rate (HR)—related metrics such as exercise heart rate (HRex) and maximum heart rate (HRmax) are widely utilized (47). HRex serves as a crucial indicator for assessing training adaptation and fatigue status. Monitoring the overall load in rugby players has shown that adult players' average heart rate during training or matches typically ranges from 75%–86% of their HRmax (17). This intensity level was defined as sub-maximal training. Importantly, these findings align with observations from other team sports, such as football, where similar heart rate responses have been documented during training and competition (48). This consistency further supports the practicality of using HRex as a reliable indicator for controlling training intensity.

In training practice, the setting and monitoring of exercise intensity are usually based on the %HRpeak method. However, heart rate is subject to interference from various factors, such as submaximal heart rate, which can vary by up to 6.5% daily (12). Therefore, when classifying low-, moderate-, and high-intensity training, other load monitoring indicators (such as blood lactate concentration) need to be integrated. Studies have shown that there is a significant correlation between high-intensity training time and changes in aerobic performance. In appropriate load training, a decrease in heart rate is usually associated with an improvement in aerobic capacity (49). However, in team sports, a decrease in players' heart rate during preseason training does not reflect fatigue but may be related to changes in plasma volume caused by training or the environment (50) Therefore, it is necessary to quantify training load and assess the physiological load of training by using a combination of multiple indicators (10, 41, 50).

Training impulse (TRIMP) is a heart rate-based method for quantifying training load, calculated by training duration × training intensity based on average heart rate, which can assess athletes' physiological stress levels (51). To accurately assess the load of interval training, the TRIMP algorithm has continued to evolve and has been combined with values such as lactate threshold and ventilatory threshold to optimize the assessment method. Several studies have used different TRIMP methods to assess the internal load in rugby training and matches and have confirmed that this method can establish a training dose-response relationship between training load and changes in maximum oxygen uptake (VO2max) (52).

In addition, the individualized TRIMP (iTRIMP) method is calculated based on individual lactate threshold. iTRIMP calculates the relationship between each person's HR changes and blood lactate concentration, monitors heart rate in real-time during training or matches, and weights it according to the relationship previously established by the athlete. This breaks through the limitations of the universal division of traditional heart rate zones and achieves precise quantification of individual-specific responses (17). Studies have shown that iTRIMP can accurately reflect the speed changes of professional youth football players at a lactate concentration of 2 mmol·L^−1^ (12). In rugby training monitoring, iTRIMP also shows good applicability, especially in different training modes such as simulated matches, physical conditioning, technical and tactical training, speed training, and strength training, with high application value (53).

#### 4.2.3 Monitoring of heart rate after exercise

Heart rate recovery (HRR) refers to the rate of decline in heart rate after exercise stops and is an indicator for assessing athletes' autonomic nervous function and training status (12). Borressen and Lambert's (54) study found that when athletes maintain the same load training, HRR remains stable; increasing the training load slows down HRR, while reducing it can accelerate HRR recovery. HRR is usually calculated between 30 s and 2 min, with the most commonly used method being the difference between immediately after exercise and 60 s later. Studies have found that the higher the exercise intensity and the higher the peak heart rate, the faster the initial recovery speed (12). However, as exercise intensity increases, the heart rate recovery speed slows down, which may be related to the sustained activation of the sympathetic nerve. The impact of exercise duration on HRR is less than that of exercise intensity. Under the same internal training load, the HRR recovery speed of high-intensity short-duration training is slower than that of low-intensity long-duration training (55), indicating that high-intensity training leads to elevated heart rates and longer recovery times, and the body has not fully adapted yet.

Scott (47) conducted a two-week experiment on rugby players, testing HRR at 1 and 2 min after exercise. The daily fluctuation range under different exercise intensities and durations was ±4.6 and ±4.1 beats, respectively, showing good consistency. Borresen (54) further pointed out that to ensure accurate assessment of HRR, testing should be conducted after high-intensity training, with exercise intensity reaching 86%–93% of maximum heart rate. Although HRR can provide more precise monitoring effects after high-intensity training, for athletes who have accumulated a certain degree of fatigue, high-intensity training may increase the risk of injury or exacerbate fatigue. Therefore, the timing of testing needs to be carefully selected in practical applications (56).

### 4.3 Heart rate variability

Heart Rate Variability (HRV) is an important biomarker for assessing the function of the autonomic nervous system. Its physiological mechanism stems from the combined regulatory effects of the sympathetic nervous system (SNS) and the parasympathetic nervous system (PNS) on sinoatrial node activity. This neural regulatory mechanism has led to the widespread use of HRV in sports science for monitoring training load and assessing functional status (57). By quantifying the fluctuation characteristics of successive R-R intervals, HRV reflects the dynamic balance between the sympathetic and parasympathetic nervous systems (57). Currently, the analysis methods for HRV mainly include time-domain analysis, frequency-domain analysis, and nonlinear dynamics analysis, which provide multidimensional assessment tools for a deeper understanding of the functional state of the autonomic nervous system.

Among the time-domain parameters based on successive R-R interval sequences, the Root Mean Square of Successive Differences (RMSSD), which is mediated by the parasympathetic nervous system, has become a core indicator for monitoring recovery in athletes due to its high sensitivity to acute fatigue states (43). In rugby, 1 min RMSSD measurements have shown high reliability in a resting state (ICC = 0.96, CV = 3.99%), allowing for a stable reflection of athletes' physiological and psychological states (58). Changes in HRV have been used to monitor the training load of rugby players. In the early stages of high-intensity training, HRV in elite rugby players typically decreases but gradually stabilizes as training adaptation improves, indicating that athletes can better maintain cardiac autonomic balance (45). In a 10-week training observation, it was found that post-match fatigue and recovery capacity showed a downward trend, and HRV usually recovered on the day after the match (MD + 1), faster than muscle soreness. This difference may stem from different types of fatigue recovery mechanisms (59, 60).

In sports training monitoring, the relationship between HRV-related indicators and training load is not linear, and an increase in training load does not necessarily lead to changes in HRV (56). For example, in pre-season high-temperature training for AFL players, vagal-related HRV parameters (such as SD1) were significantly correlated with RPE-TL scores (r ≈ 0.5) (50). However, this change in parasympathetic activity may partly originate from thermoregulation or changes in plasma volume, rather than directly reflecting training adaptation or fatigue status (50, 58). After endurance training in elite football players, HRV briefly decreased, but in subsequent studies, it was found that HRV did not change significantly within the season week (50).

HRV indicators reflect athletes' adaptive responses to training stimuli, rather than simply reflecting changes in training load (61). If HRV indicators fluctuate significantly in elite rugby players during changes in training load, then their sports performance should be closely monitored. Especially in the run-up to important matches, significant fluctuations in HRV may indicate poor autonomic nervous system adaptability, leading to cumulative fatigue or performance decline (44, 61). This indicates that HRV feedback on training load is influenced by multiple factors, including training intensity, environmental conditions, and individual differences.

The variation in HRV during the post-exercise recovery phase is influenced by a multitude of factors, including blood pressure regulation, feedback from exercise intensity, and post-exercise metabolic stimulation (42). These factors collectively contribute to the reduction of sympathetic nervous activity and the enhancement of parasympathetic activity. Higher HRV values post-exercise are indicative of positive adaptation and superior recovery status, whereas lower values suggest fatigue and suboptimal recovery.

To minimize data error, it is recommended that HR be monitored as a training load index using weekly averages rather than single measurements (16, 61). However, in team sports, monitoring HR indices based on multi-day averages faces several challenges, including data collection, data analysis, individual differences, and the dynamic adjustment of training programs. These factors limit the practical application of HR in team sports (40).

## 5 Load monitoring based on biochemical indicators

After training, the metabolic level of the human body changes. Monitoring biochemical indicators in athletes helps to assess their training load, recovery status, muscle damage, and inflammatory response. In rugby-related studies, creatine kinase (CK) and myoglobin (Mb) are commonly used for assessment, while C-reactive protein (CRP) is often used as a biomarker for inflammatory response (21). In addition, biomarkers related to neuroendocrine function, such as cortisol and testosterone, are closely related to fatigue and recovery status after matches.

### 5.1 Blood indicators

#### 5.1.1 Blood lactate

Blood lactate (BLA) is one of the main indicators for assessing the training intensity of athletes, peaking about 3 min after exercise ends, and is therefore commonly tested immediately after training. Studies in rugby have shown that the peak BLA for male athletes is 2.9–20.2 mmol·L^−1^, while for female athletes it is 3.4–14.6 mmol·L^−1^ (62). Moreover, the clearance rate of BLA after exercise can reflect the body's recovery capacity; a faster clearance rate indicates better training status and aerobic capacity, while a slower rate suggests possible accumulation of fatigue or overtraining (63).

BLA concentration, often measured alongside heart rate and oxygen uptake, provides a comprehensive assessment of athletes' aerobic and anaerobic capabilities. In rugby, high-intensity activities like sprints rely primarily on anaerobic metabolism and cannot be sustained by aerobic metabolism alone (1). For example, Granatelli (64) reported BLA concentrations of 8.7 (1.7) mmol·L^−1^ 2 min after halftime and 11.2 (1.4) mmol·L^−1^ 2 min after an international match. Similarly, Couderc (65) found a BLA concentration of 11.7 ± 3.7 mmol·L^−1^ following a sevens rugby match, aligning with Granatelli's findings (11.2 ± 1.4 mmol·L^−1^) (64).

Post-match, the peak BLA concentration for sevens rugby players was approximately 16.3 (2.4) mmol·L^−1^, whereas for fifteens rugby matches, the BLA concentration ranged from 4.8–7.2 mmol·L^−1^ (62, 66). These data indicate that glycolysis plays a significant role in the energy supply of rugby players, and athletes need to possess a high capacity for acid tolerance to cope with high-intensity activities in successive matches (67) (Table 5).

**Table 5:** Summary of key findings on training load in rugby players using biochemical indicator-based monitoring technologies.

Authors	Year	Study objectives	Main results	Reference
Banfi et al.	2006	This study assessed haematological parameters in elite rugby players throughout a competitive season.	The study found that training and competition workload significantly influenced these parameters, with reticulocytes and soluble transferrin receptor being the sensitive indicators for studying iron metabolism in rugby players.	(67)
Cunniffe et al.	2010	This study evaluated the time course of immune and endocrine changes following an international rugby game. Significant increases in cortisol, IL-6, and creatine kinase were observed, along with decreases in T lymphocytes and neutrophil function, indicating immune suppression.	The study suggests that rugby games cause significant physiological stress, with disturbances in immune function lasting up to 38 h post-game. Monitoring these biochemical markers can help assess recovery and training load in elite rugby players.	(72)
Gaviglio and Cook	2014	The study aims to assess the response of salivary-free testosterone and cortisol concentrations across midweek skill-based training sessions and investigate their association with subsequent match outcomes in professional rugby union players.	Midweek T/C ratio measurements in response to skill-based training sessions show potential as an early indicator of subsequent successful performances. Monitoring these hormonal changes may assist in assessing competitive readiness leading up to matches.	(78)
Lindsay et al.	2015	This study investigates the positional demands of professional rugby and their impact on psychophysiological stress using biochemical indicators such as urinary neopterin, cortisol, and salivary immunoglobulin A.	Results show that while distance covered and number of impacts vary by position, changes in stress markers are independent of position. This highlights the need for position-specific training and recovery strategies to manage player load effectively.	(84)
Lindsay et al.	2015	This study assessed the effectiveness of selected biomarkers in measuring acute and cumulative physiological stress in professional rugby union.	Significant changes were observed in all markers, indicating high levels of muscle damage, psychophysiological stress, and immune system suppression. The study demonstrates the potential of these non-invasive biomarkers for individualized stress assessment in rugby players.	(96)
Bouaziz et al.	2016	This study examined the effectiveness of biochemical indicators in monitoring training load in elite rugby sevens players.	Urinary C and Cn levels rose during intensive training while A and NA decreased, with the C/Cn ratio strongly correlating to training load and fatigue; all biomarkers normalized post-tapering, validating this ratio as a practical monitoring tool for rugby athletes.	(93)
Gonzalez et al.	2020	This study evaluated the release dynamics of muscle damage markers (creatine kinase, lactate dehydrogenase, and aspartate aminotransferase) in saliva samples after rugby sevens matches and analyzed gender differences.	While CK and LDH did not show significant changes, AST levels increased significantly after the final match. Men exhibited higher enzyme levels than women, indicating greater muscle damage.	(69)
Tiernan et al.	2020	Investigate the relationship between salivary cortisol, training load, and subjective markers of recovery in elite Rugby Union players.	Salivary cortisol can be used as an objective marker to assess recovery status at the beginning of the week and preparation for matches. Combining salivary cortisol monitoring with subjective markers and planned training load can help optimize individual player performance and recovery.	(83)
Aben et al.	2022	This systematic review examines the biochemical responses to rugby match-play, focusing on CK and C concentrations.	These biochemical markers indicate significant muscle stress and fatigue, highlighting the importance of monitoring CK and C to assess recovery and manage training loads effectively in rugby players.	(70)
James et al.	2023	This study investigates the blood lactate responses of male and female rugby sevens players across an international tournament.	Results show high postmatch blood lactate concentrationswith significant decreases only after the final match, these findings highlight the substantial metabolic demands of rugby sevens and the need for individualized anaerobic training prescriptions and recovery strategies to enhance players' metabolic capacity and performance across multiple matches.	(62)

CK, creatine kinase; C, cortisol; Cn, cortisone; IL-6, Interleukin-6; LDH, lactate dehydrogenase; AST, aspartate aminotransferase; A, adrenaline; NA, norepinephrine.

#### 5.1.2 Muscle damage indicators

Blood biochemical monitoring, as a core means of assessing athletes' functional status, can precisely quantify the relationship between training intensity, match load, and recovery process through periodic sampling (68). The standardized blood sampling procedure requires subjects to be in a fasting state and complete sampling between 8:00 and 9:00 in the morning (41).

When assessing the physiological function of rugby players, CK, aspartate aminotransferase (ASAT), and alanine aminotransferase (ALAT) are key indicators for evaluating muscle damage. CK is widely distributed in skeletal muscle, myocardium, and brain tissue, directly participating in muscle contraction and energy metabolism, with its level changes highly correlated with the degree of muscle fatigue. ASAT and ALAT also significantly increase after training or matches, and these indicators are highly sensitive to high-load training. Their concentration changes can not only monitor the liver function status of athletes but also assess muscle recovery (24, 67).

CK, a key enzyme in skeletal muscle energy metabolism, significantly increases following eccentric exercise and external impacts (68, 72). In rugby, CK levels rise sharply 30 min post-match, peak at 12–36 h (a 265.5% increase from baseline), and remain elevated at 72 h (21, 50, 69). However, some studies suggest that the reported 12–16 h “peak” may be due to a lack of measurements beyond 24 h, and recommend extending sampling times for more accurate assessments (70). Forwards, who experience more physical contact, show a significantly higher CK increase than backs (24 h: 285.1% vs. 167.9%; 48 h: 144.6% vs. 88.9%) (71). This highlights the specific distribution of sport-specific load fatigue. While long-term CK assessments (beyond 4 days) aid in understanding recovery, individual differences should be noted (70).

In addition, the resting CK levels of rugby players are significantly higher than those in other contact sports (such as football and basketball), and this difference may stem from adaptive changes to cumulative fatigue caused by frequent collisions over the long term (72). Therefore, particular attention should be paid to technical training, including collisions, in training to avoid injuries and overtraining (73).

#### 5.1.3 Immune indicators

Changes in blood components such as C-reactive protein, hemoglobin, white blood cells, and neutrophils can be systematically monitored during high-intensity matches and training to scientifically assess athletes' recovery status and health levels (17, 72). Studies have shown that hemoglobin concentration often changes during training, especially after high-intensity exercise (67). In the early stages of training, changes in hemoglobin concentration are mainly due to an increase in plasma volume, leading to hemodilution. However, as high-intensity training continues, erythropoiesis is inhibited (6, 68). This biphasic change may reduce blood oxygen-carrying efficiency, thereby affecting athletic performance.

White blood cell (WBC) and its subpopulation analysis reveal the dynamic balance mechanism of immune function. The increase in total WBC count after matches or high-intensity training is mainly due to a significant increase in neutrophils (PMN), accounting for 70% of the increase in WBC (72). This change is usually associated with muscle fatigue, inflammatory response, and recovery process induced by exercise. However, despite the increase in WBC and neutrophil counts, their functional capacity may not be enhanced synchronously. Studies have shown that in continuous rugby exercise without adequate recovery, although an increase in cell numbers is observed, the function of neutrophils (such as degranulation) significantly decreases (−65%) (7). Monitoring the changes in WBC and neutrophil counts can effectively assess whether athletes have overtraining or other health issues.

C-reactive protein (CRP) levels are compared immediately after matches to baseline levels, showing an increase of up to 9.8% (74). Further studies indicate that CRP rises to 64.1% and 121.6% of baseline at 16 and 24 h post-match, respectively, peaking at 201.0% of baseline within 24–48 h and returning to baseline within 72 h (21). CRP appears more sensitive than creatine kinase (CK) in reflecting muscle fatigue and recovery related to collisions (23). Additionally, a decrease in plasma glutamine levels and an increase in glutamate levels reduce the glutamine-to-glutamate ratio. A ratio below 3.58 suggests increased training load (75). This decline in glutamine may be linked to increased gluconeogenesis due to glycogen depletion, which promotes greater amino acid uptake (76). However, limited research connects glutamine reduction to impaired immune function (73), so further studies are needed to confirm the utility of the glutamine-to-glutamate ratio as a fatigue marker.

Interleukin-6 (IL-6), an inflammatory cytokine, has been extensively studied as a marker of fatigue and overtraining. IL-6 levels significantly increase following exercise, particularly after high-intensity and prolonged activities, with its elevation being closely associated with muscle damage and inflammatory responses (72). Research indicates that the rise in IL-6 not only reflects the extent of muscle damage but may also play a role in modulating immune responses and facilitating the recovery process (72). Therefore, monitoring IL-6 levels can provide valuable insights into athletes' training loads and recovery status.

#### 5.1.4 Hormonal indicators

Hormonal indicators play a crucial role in assessing athletes' endocrine status, their adaptation to training load, and their recovery status. Testosterone (T) and cortisol (C) serve as critical biomarkers for evaluating training adaptation and recovery. T mediates anabolic processes including protein synthesis and glycogen replenishment (77). Post-exercise T responses indicate load appropriateness: unchanged levels suggest insufficient stimulus; 10%–20% decreases reflect optimal adaptation; while sustained drops >25% signal excessive load requiring intervention (78). These hormonal patterns enable precise training load management to prevent overtraining.

C levels are closely tied to training load, increasing during both endurance and resistance training (8). It reflects both physiological stress from high-intensity exercise and psychological stress from competitive environments (79). Before rugby matches, excitement, anxiety, and stress can alter cortisol and testosterone levels. During training, decreased testosterone and increased cortisol often indicate an imbalance between anabolic and catabolic processes (80). Post-match, cortisol may rise by 30.0%–52.5%, and testosterone may drop by about 43%, reducing the testosterone-to-cortisol (T/C) ratio significantly. This ratio continues to decline for up to 36 h and remains elevated for up to five days (17, 23, 72), aligning with findings in elite rugby (81). A significant increase in the T/C ratio suggests the body's response to high training loads by boosting testosterone to aid muscle repair and synthesis (72).

In Dubois's study (23), the focus was on the changes in insulin-like growth factor (IGF-1). The ratios of these indicators (such as the T/C ratio and IGF-1/C ratio) provide insights into fatigue and recovery in rugby players. IGF-1 shows high sensitivity to training and match loads (50). The accumulation of long-term training load correlates positively with IGF-1 levels, reflecting the recovery and regeneration processes following muscle fatigue. Changes in the IGF-1/C ratio and T/C ratio can serve as indicators of training status and help monitor non-functional overreaching (68, 82).

### 5.2 Saliva

#### 5.2.1 Salivary secretory immunoglobulin A

Saliva, as a non-invasive biological sample, offers several advantages such as ease of collection, safety, and cost-effectiveness. Its protein content is lower compared to serum (80). Salivary secretory immunoglobulin A (S-IgA) is one of the key indicators for assessing the immune system health of athletes. Studies have shown that in rugby players, S-IgA secretion rates decrease from an average of 401.3 µg/min pre-match to 298.3 µg/min post-match, with variations observed among players in different positions (50, 83, 84). Monitoring changes in S-IgA can help assess the immune function of rugby players during high-intensity training or matches, thereby revealing the impact of exercise intensity on the immune system (Table 5).

#### 5.2.2 Salivary cortisol and testosterone concentrations

The concentrations of cortisol and testosterone in saliva accurately reflect the adrenal cortex's adaptive response to exercise intensity. As exercise intensity and duration increase, the rise in cortisol levels becomes more pronounced (84). Monitoring before and 30 min after matches shows an increase in cortisol levels, peaking 30 min post-match (85). It is important to note that pre-match cortisol levels also reflect the athlete's psychological state, influenced by cognitive expectations and perceived anxiety, often used to regulate pre-match excitement (70). Therefore, the significant post-match increase in cortisol may be influenced by psychological factors and differences in exercise type and duration.

#### 5.2.3 Enzyme indicators

Regarding the assessment of muscle fatigue, there is currently debate over the changes in enzyme indicators such as CK, aspartate aminotransferase (AST), and lactate dehydrogenase (LDH) in saliva before and after matches. González (69) found no significant differences in CK and LDH concentrations before and after matches, despite high RPE according to the Borg scale. In contrast, Barranco (86) observed significant changes in these enzyme indicators 30 min and 12 h post-match, with levels remaining elevated up to 24 h afterward (80). This discrepancy may be due to differences in the timing of sample collection, indicating a delay in the transfer of these enzymes from blood to saliva.

Therefore, it is essential to consider the cumulative effects of exercise load and the timing of sample collection. Changes in salivary enzyme indicators typically become apparent only after a certain period of load accumulation. For instance, significant changes in salivary AST levels were observed only after four weeks of aerobic exercise intervention (87). This suggests that changes in salivary enzyme indicators are time-dependent.

Studies in football have shown that salivary CK and LDH levels increase 30 min post-match, while AST levels remain unchanged, possibly due to lower sensitivity of AST in saliva (86). In rugby research, male players exhibited higher CK and LDH levels before and after matches, with LDH being the only enzyme showing no gender differences (69, 83), indicating potential gender differences in enzyme responses. Further research has shown that physiological responses to exercise also vary by gender. For example, females exhibit higher heart rates and RPE levels post-exercise, while males show more pronounced declines in strength, suggesting that females may have better fatigue resistance when facing the same load (88). Additionally, studies have indicated that females have a lower incidence of muscle damage and a more gradual increase in serum CK levels after injury (89), highlighting the need to consider gender differences when assessing salivary enzyme indicators.

### 5.3 Urine

#### 5.3.1 Hormonal indicators

Urine markers serve as crucial tools for assessing athletes' health and metabolic status, operating within the domain of metabolomics. By examining fluctuations in metabolites, these markers uncover the fundamental sources of endogenous substances and energy supply within the body during exercise. Monitoring the dynamic changes in metabolic biomarkers post-exercise aids in elucidating the patterns of potential biological indicators associated with exercise (90) (Table 5).

The cortisol/corticosterone (C/Cn) ratio and adrenaline/noradrenaline (A/NA) ratio, reflecting HPA and SAM axis activity, are key indicators of athletes' stress and recovery (91, 92). These hormones show significant changes in endurance sports like swimming and running (93). High-intensity training increases C, Cn, and the C/Cn ratio by +27%, +13%, and +22%, respectively, while A and NA levels drop by −30% and −25% (91, 93). These markers return to baseline after a two-week recovery. NA is mainly affected by physical stress, while A is more influenced by psychological stress (94). Monitoring training load should include both physiological and psychological aspects, with regular use of the Session-RPE test to prevent overtraining (31, 91).

Despite temporary disturbances in homeostasis during high-intensity training, levels of C, Cn, and the A/NA ratio return to baseline after a brief adjustment period, coinciding with performance improvement. This recovery period facilitates performance enhancement through supercompensation, further underscoring the importance of adequate recovery in preventing overtraining. Overall, levels of C, Cn, A, and NA and their ratios, particularly the C/Cn ratio, in urine serve as effective biomarkers for monitoring changes in training load, fatigue, and performance. Changes in the SAM and HPA axes reflect athletes' physiological adaptation to long-term training, offering a multi-faceted basis for monitoring the training of elite athletes.

#### 5.3.2 Metabolite indicators

Urine metabolites and physicochemical parameters can indicate exercise-induced bodily changes. In rugby players, post-match urine creatinine levels rise significantly, reflecting muscle metabolic function (93). Urine specific gravity (SG), a measure of urine concentration and hydration status, also increases. The World Anti-Doping Agency (WADA) suggests using SG to assess overhydration (SG > 1.030) and dehydration (SG < 1.003). Additionally, other parameters of urine samples, such as urine color and osmolality, also have certain reference value for understanding athletes' hydration status (41).

In rugby, there is a significant decrease in urine pH from pre-match to post-match (*P* = 0.002), dropping from 6.33 ± 0.6–5.67 ± 0.36 (95, 96). This change indicates that high-intensity exercise may lead to the accumulation of acidic metabolic products in the body, thereby affecting the acid-base balance of urine.

In their study on rugby union, Lindsay (96) observed that, after correction for urine specific gravity, levels of neopterin increased 1.75-fold and total neopterin increased 2.3-fold. These markers exhibited a significant post-game increase, returning to baseline within 17 h. Their temporal changes suggest that neopterin and total neopterin can serve as novel indicators for detecting acute inflammatory responses, particularly in the context of physical exercise (96). It is important to note that individual athletes exhibited substantial variability in their responses to game-related stress, underscoring the critical importance of personalized monitoring.

Similar to hormonal changes, urinary myoglobin effectively quantifies exercise load, with post-match levels (17.5 ± 12.6 ng/ml) significantly exceeding pre-match values (6.2 ± 5.7 ng/ml; *p* < 0.05, *η*² = 0.353) and normalizing within 17 h (6.8 ± 6.9 ng/ml) (95). As a sensitive muscle fatigue marker, its plasma/urine concentrations correlate with collision frequency—professional rugby matches elevate levels from <5 ng/ml pre-match, with greater increases after consecutive games (96, 97). Variability between matches depends on competition level, sampling timing, and individual differences (69, 96, 97), necessitating pH adjustment and standardized analysis timing for reliable measurements.

## 6 Limitations of internal and external load monitoring

The assessment of athletes' training load necessitates integrated monitoring of both external and internal loads to fully capture training stimuli and physiological responses. External load metrics, such as distance covered, sprint frequency, and collision intensity, effectively quantify physical demands but inherently fail to directly reflect cumulative fatigue states. Conversely, internal load measures, while providing crucial physiological insights, are complicated by significant inter-individual variability in adaptation patterns, thereby complicating interpretation. This dual-monitoring necessity is particularly pronounced in rugby, where positional specialization and individual response differences create complex load-adaptation relationships that demand personalized monitoring frameworks.

Considerable promise in detecting subtle load-fatigue-injury relationships through advanced pattern recognition, several nuanced limitations persist. First, ecological validity constraints mean that current monitoring tools often struggle to capture rugby-specific movement patterns, such as contested rucks and scrum engagements, with sufficient granularity, potentially underestimating position-specific mechanical loads. Second, temporal dissociation between acute workload metrics and delayed physiological responses further complicates monitoring accuracy, particularly for neuromuscular and endocrine adaptations that may require 48–72 h to fully manifest. Third, the intervention paradox suggests that intensive monitoring may inadvertently modify player behavior, such as subconsciously avoiding high-intensity efforts during GPS-monitored sessions, thereby distorting the “true” load representation. Fourth, data integration challenges arise because machine learning models require exceptionally clean, synchronized multimodal datasets, yet rugby's chaotic environment often results in missing data, such as dislodged GPS units and failed blood sampling, which can compromise model robustness. Finally, positional generalization limits indicate that algorithms trained predominantly on forward-dominated cohorts often fail to accurately predict backline injury risks due to different load-fatigue profiles.

Despite these constraints, multifactorial/multimodal models, particularly those leveraging machine learning, represent a significant advancement by simultaneously analyzing biomechanical load patterns, physiological response trajectories, and biochemical marker fluctuations. These advanced models can identify complex, non-linear relationships that traditional approaches often overlook, particularly in predicting non-contact injury risks. However, their practical implementation in rugby remains constrained by the sport's unique demands, necessitating ongoing refinement of position-specific monitoring protocols and validation against rugby-specific outcomes. Future research should focus on developing standardized protocols for sensor deployment during contact situations and improving the temporal alignment of multimodal data streams to enhance the accuracy and reliability of load monitoring in rugby.

## 7 Summary

Monitoring, automated technologies-such as GPS and microsensor systems-and Time-Motion Analysis (TMA) have furnished detailed data to elucidate athletes' movement patterns. These technologies can capture key parameters like athletes' positions, speeds, accelerations, and directional changes in real-time, thereby accurately quantifying the external stimuli of training and matches. However, these methods still encounter certain challenges in practical applications. For instance, key technical standards, including sensor placement and impact force threshold definition, have yet to be standardized. Additionally, traditional monitoring technologies are incapable of fully quantifying specific movements in rugby, such as “isometric efforts”, which somewhat limits the accuracy and comprehensiveness of external load monitoring.

Internal load monitoring centers on evaluating athletes' physiological and psychological states. Subjective perceived intensity, exemplified by the session-RPE scale, captures athletes' subjective feedback on training intensity. Meanwhile, heart rate-related indicators-encompassing resting heart rate, exercise heart rate, heart rate recovery, and heart rate variability-unveil athletes' fatigue status and autonomic nervous function changes from a physiological standpoint. For example, heart rate variability (HRV), a pivotal biomarker for assessing autonomic nervous system function, can reflect athletes' adaptation to training load through its dynamic fluctuations. However, the efficacy of heart rate monitoring has not reached a consensus across different studies. Moreover, HRV indicators reflect athletes' comprehensive adaptive responses to training stimuli, rather than changes attributable to a singular training load.

Load monitoring predicated on biochemical indicators offers robust support for a deeper comprehension of athletes' physiological status. Multidimensional biochemical monitoring methods, which include blood indicators (such as blood lactate, muscle damage markers, immune indicators, and hormonal indicators), saliva indicators (such as salivary secretory immunoglobulin A, cortisol, and testosterone concentrations), and urine indicators (such as hormonal indicators, metabolite indicators, and myoglobin), can provide a comprehensive assessment of athletes' metabolic levels, muscle damage, inflammatory responses, and endocrine status. These biochemical indicators not only aid in quantifying training load but also furnish a scientific basis for tailoring training programs. However, their collection and analysis processes are relatively intricate, and some indicators (such as salivary enzyme indicators) are sensitive to sampling time, with potential delays in their changes.

Despite the availability of various methods to quantify the load of rugby training and matches, no unified gold standard measurement method has been established. The complexity of rugby further exacerbates this issue, with total load being influenced by a confluence of external and internal factors. When selecting methods to quantify athletes' load, it is imperative to comprehensively consider the characteristics of the activity, the feasibility, stability, and accuracy of the measurement methods, and ensure that the data holds practical reference value for coaching and research practice. It is recommended to combine external and internal measurement methods to achieve a more comprehensive assessment of load.

To effectively monitor the training load and fatigue status of rugby players, multiple indicators should be systematically tracked from the perspective of daily training or the entire season. This systematic monitoring approach can more accurately enhance training effectiveness, ensure that athletes maintain optimal competitive form during matches, and better adapt to the actual demands of competition.
